# miR-93 Promotes Cell Proliferation in Gliomas through Activation of PI3K/Akt Signaling Pathway

**DOI:** 10.18632/oncotarget.3221

**Published:** 2015-03-13

**Authors:** Lili Jiang, Chanjuan Wang, Fangyong Lei, Longjuan Zhang, Xin Zhang, Aibin Liu, Geyan Wu, Jinrong Zhu, Libing Song

**Affiliations:** ^1^ Department of Pathophysiology, Guangzhou Medical University, Guangzhou 510182, China; ^2^ State Key Laboratory of Oncology in Southern China, Department of Experimental Research, Cancer Centre, Sun Yat-sen University, Guangzhou 510060, China; ^3^ Department of the Central Laboratory, The First Affiliated Hospital/School of Clinical Medicine of Guangdong Pharmaceutical University, Guangzhou 510080, China; ^4^ Laboratory of Surgery, First Affiliated Hospital, Sun Yat-sen University, Guangzhou 510080, China; ^5^ Department of Biochemistry, Zhongshan School of Medicine, Sun Yat-sen University, Guangzhou, Guangdong 510080, China

**Keywords:** PI3K/Akt, miR-93, gliomas

## Abstract

The PI3K/Akt signaling pathway is frequently activated in various human cancer types and plays essential roles in development and progression of cancers. Multiple regulators, such as phosphatase and tensin homolog (PTEN) and PH domain leucine rich repeat protein phosphatases (PHLPP), have also found to be involved in suppression of the PI3K/Akt signaling pathway. However, how suppressive effects mediated by these regulators are concomitantly disrupted in cancers, which display constitutively activated PI3K/Akt signaling, remains puzzling. In the present study, we reported that the expression of miR-93 was markedly upregulated in glioma cell lines and clinical glioma tissues. Statistical analysis revealed that miR-93 levels significantly correlated with clinicopathologic grade and overall survival in gliomas. Furthermore, we found that overexpressing miR-93 promoted, but inhibition of miR-93 reduced, glioma cell proliferation and cell-cycle progression. We demonstrated that miR-93 activated PI3K/Akt signaling through directly suppressing PTEN, PHLPP2 and FOXO3 expression via targeting their 3′UTRs. Therefore, our results suggest that miR-93 might play an important role in glioma progression and uncover a novel mechanism for constitutive PI3K/Akt activation in gliomas.

## INTRODUCTION

The phosphatidylinositol-3-OH kinase (PI3K)-Akt pathway is a major signaling cascade that is activated in a large variety of human cancers [1]. Phosphatidylinositol-3, 4, 5-trisphosphate (PIP3), the enzymatic product of the PI3K, recruits the serine-threonine kinase Akt through binding to its pleckstrin-homology (PH)-domain [1, 2], which releases the PH domain from masking the kinase domain and thus catalyzes phosphorylation of Akt on Thr308 in the activation loop and on Ser473 in the carboxylterminal hydrophobic motif [3–5]. The phosphorylation of Akt activates downstream target genes involved in survival, proliferation, cell cycle progression, growth and migration of tumor cells, as well as angiogenesis [1–4, 6, 7]. For example, Akt could phosphorylate and suppress the transcriptional factor FOXO3, leading to upregulation of Cyclin D1 and suppression of p27^Kip1^ that promote cell cycle progression and cell proliferation [8, 9].

On the other hand, multiple negative regulators have been found to be involved in the deactivation of PI3K/Akt signaling, such as Phosphatase and tensin homolog (PTEN) and The PH domain leucine rich repeat protein phosphatases (PHLPP). Function as tumor suppressor [10–13], PTEN could inhibit PI3K/Akt signaling through reversing the phosphorylation of PIP3, returning it to PIP2, result in antagonizing the function of PI3K [14]. Mutation or deletion of PTEN results in increased cell proliferation and reduced cell death [14] and have been found in more than 50% of glioblastomas and in 60–80% of glioma cell lines [15–19]. PHLPP2, an isoform of PHLPP phosphatases, has been reported to induce cell cycle arrest and apoptosis and suppress tumor growth by directly dephosphorylating and inactivating of Akt at Ser473 specifically and depressing the activity of PI3K/Akt signaling pathway subsequently [20, 21]. Loss of function of PHLPP2 might result in tumorigenesis and tumor progression. Therefore, understanding the precise molecular mechanisms underlying regulation of negative regulators of PI3K/Akt signaling could provide new insights into the pathogenesis of cancers and lead to more effective anticancer therapy strategies.

MicroRNAs (miRNAs), small non-coding RNAs of 20–22 nucleotides, are involved in multiple biological processes, such as cellular differentiation, proliferation, oncogenesis, angiogenesis, invasion, and metastasis [22–25]. It has been demonstrated that miRNAs play important roles during human cancer progression through negatively regulating their target mRNAs via recognizing and binding the 3′ untranslated region (3′UTR) of the mRNAs in a sequence-specific manner [26, 27]. The post-transnationally regulation function of miRNAs supports novel sights of tumor suppressive genes expression. Further, in view of the close relationship between microRNA expression and multiple biological aspects of cancer progression, miRNAs are considered as potential novel targets for anti-cancer therapies [26–28]. miR-93 was found to be markedly upregulated nasopharyngeal carcinoma and breast cancer and significantly correlated with poor prognosis [29, 30]. MiR-93 overexpression has an important role in promoting lung cancer cell growth, angiogenesis and metastasis and inhibition of miR-93 significantly suppressed HepG2 cell proliferation, migration and colony formation [31–34]. Therefore, miR-93 acts as an onco-miR and might be served as a potential anti-cancer target.

In the present study, we found that miR-93 was significantly upregulated in glioma cell lines and glioma tissues. Overexpressing miR-93 promoted, but inhibition of miR-93 reduced, glioma cell proliferation and cell-cycle progression. Furthermore, we demonstrated that miR-93 activated PI3K/Akt signaling through downregulating PTEN, PHLPP2 and FOXO3 expression via targeting their 3′UTRs. Therefore, our results suggested that miR-93 might play important roles in the development and progression of gliomas and represent as a potential therapeutic target for glioma therapy.

## RESULTS

### miR-93 is upregulated in gliomas and correlates with patient prognosis

**Figure 1:**
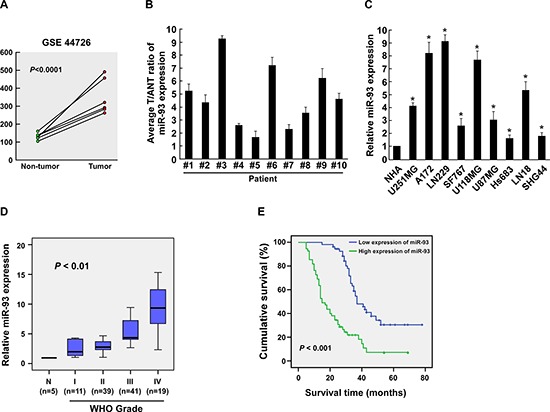
miR-93 is upregulated in gliomas **(A)** miR-93 is upregulated in paired glioma tissues and peripheral non-tumor tissues, each pair prepared from a same patient (*P* < 0.0001; NCBI/GEO/GSE44726). **(B)** Real-time PCR analysis of miR-93 expression in ten paired glioma and adjacent non-tumor tissue specimens. **(C)** Real-time PCR analysis of miR-93 expression in 9 glioma cell lines and NHA. Transcript levels were normalized by *U6* expression. **(D)** Correlation between miR-93 expression in different WHO grading of gliomas assessed by real-time PCR. Transcript levels were normalized by *U6* expression. The boundaries of the boxes represent the lower and upper quartile; lines within boxes and whiskers denote median and extremum, respectively. **(E)** Correlation between miR-93 levels and survival by Kaplan-Meier analysis of patients with low miR-93 (≤ the median, *n* = 55) or high (> the median, *n* = 55) expression. Experiments were repeated at least 3 times with similar results, and error bars represent ± SD. *P < 0.05.

By analyzing a published micro-array-based high-throughput assessment (NCBI/GEO/GSE44726), we found that miR-93 was upregulated significantly in the glioma tissues compared with the peripheral noncancerous tissues (Figure 1A). Consistently, real-time PCR analysis revealed that miR-93 was significantly overexpressed in 10 freshly collected gliomas samples as compared with paired adjacent non-tumor tissues obtained from the same patient (Figure 1B), and 9 glioma cell lines compared with the normal human astrocytes (NHA) control (Figure 1C). Therefore, these results suggest that miR-93 is upregulated in gliomas.

To assess whether miR-93 upregulation is correlated with glioma progression, we further examined miR-93 expression in 110 archived clinical glioma specimens. As shown in Figure 1D, miR-93 levels remained low in tumors of grades I but became markedly higher in those at grade III and was further elevated in grade IV gliomas. Statistical analysis revealed that miR-93 levels strongly correlated with glioma WHO grade (*P* < 0.01) (Figure 1D, Supplementary Table 2). Kaplan-Meier analysis and log-rank test were employed and showed that the miR-93 levels significantly correlated with patient survival (*P* < 0.001; Figure 1E, Supplementary Table 2). High miR-93 expression was closely associated with shorter overall survival time, which suggests a possible link between high-level miR-93 expression and progression of human gliomas and highlights the potential value of the molecule as a predictive biomarker for disease outcome.

Furthermore, univariate and multivariate Cox regression analyses revealed that the expression of miR-93 and glioma grade was identified as an independent prognostic factor (Supplementary Table 3). Taken together, our results suggest that miR-93 is upregulated in glioma and might represent a novel biomarker for the progression and prognosis of patients with glioma.

### Overexpression of miR-93 promotes proliferation and cell cycle progression of glioma cells

**Figure 2:**
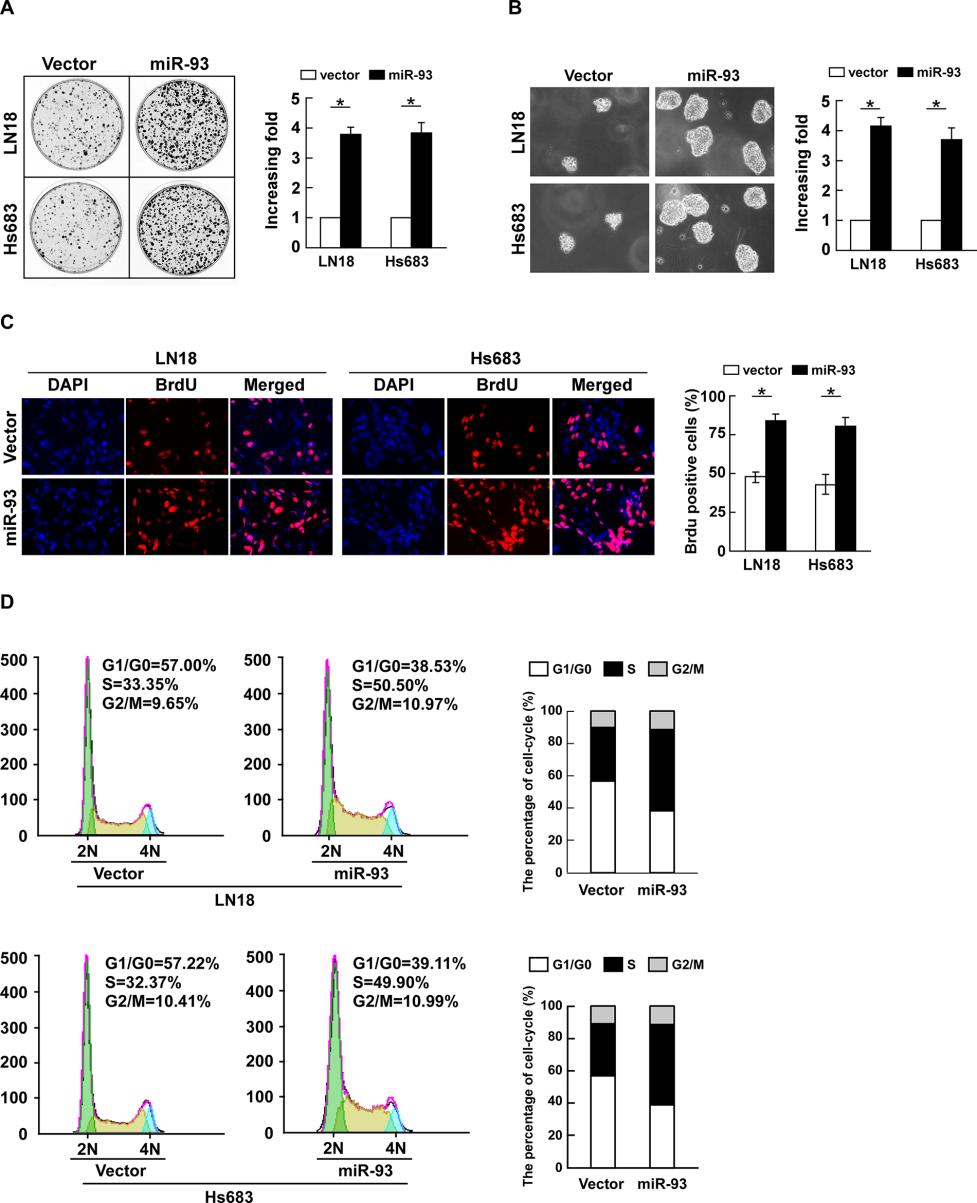
miR-93 promotes cell proliferation and cell-cycle progression in glioma cells **(A)** Representative micrographs (left) and quantification (right) of colonies formed by indicated glioma cell lines are shown 10 days after inoculation. **(B)** Effects of ectopic miR-93 on the tumorigenicity of the indicated glioma cell lines, as determined by anchorage-independent growth ability assay. **(C)** Representative micrographs (left) and quantification (right) of BrdUrd incorporating-cells of indicated glioma cells. **(D)** Effects of miR-93 overexpression on the cell cycle progression of glioma cells measured by flow cytometric analysis. Experiments were repeated at least 3 times with similar results, and error bars represent ± SD. *P < 0.05.

To investigate the biological function of miR-93 in the development and progression of glioma, glioma cells LN18 and Hs683 stably expressing miR-93 were established for the further study (Supplementary Figure 1). The result of colony formation assay revealed that ectopically expressing miR-93 in both LN18 and Hs683 cells markedly enhanced their growth ability, as indicated by the increase in colony numbers and sizes (Figure 2A). Consistently, an anchorage-independent growth assay revealed that miR-93-overexpressing LN18 and Hs683 cells formed more and larger-sized colonies than control cells (Figure 2B). Furthermore, the level of DNA synthesis, examined with BrdUrd incorporation assay, was significantly elevated in miR-93 transduced glioma cells, whereas the vector control cells displayed relatively lower BrdUrd incorporation rates (Figure 2C). Moreover, cell cycle analysis showed significant increases in the percentages of cells in the S peak while decreased percentages of cells in the G1/G0 peak (Figure 2D). Collectively, these results demonstrate that miR-93 functions to enhance proliferation, tumorigenicity and cell cycle progression of glioma cells.

### Inhibition of miR-93 attenuates proliferation and cell cycle progression of glioma cells

**Figure 3:**
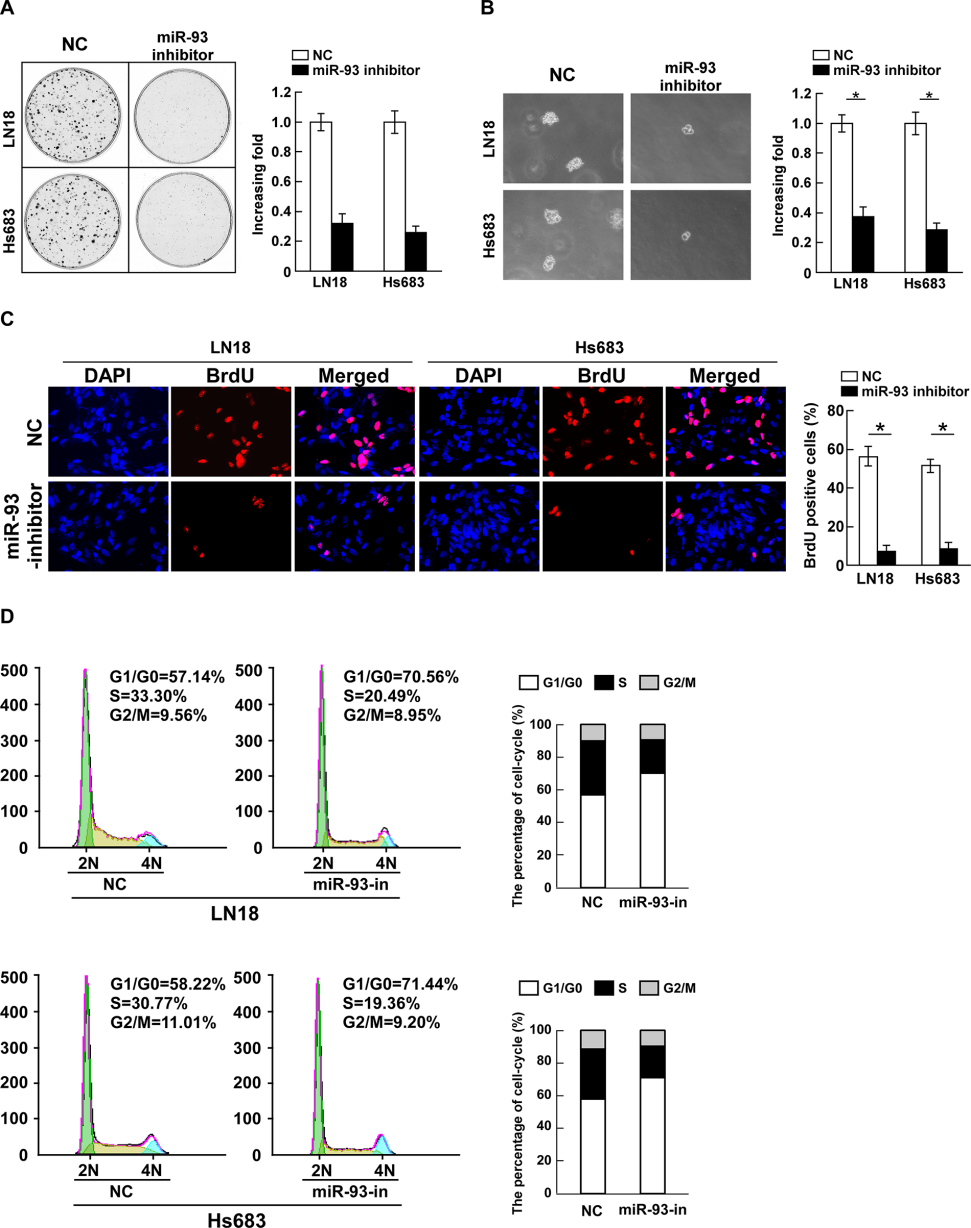
Inhibition of miR-93 reduces cell proliferation and cell-cycle progression in glioma cells **(A)** Representative micrographs (left) and quantification (right) of colonies formed by indicated glioma cell lines are shown 10 days after inoculation. **(B)** Effects of miR-93 inhibitor on the tumorigenicity of the indicated glioma cell lines, as determined by anchorage-independent growth ability assay. **(C)** Representative micrographs (left) and quantification (right) of BrdUrd incorporating-cells after transfection with miR-93 inhibitor or NC. **(D)** Effects of miR-93 inhibitor on the cell cycle progression of glioma cells measured by flow cytometric analysis. Experiments were repeated at least 3 times with similar results, and error bars represent ± SD. *P < 0.05.

Loss-of-function studies using a miR-93 inhibitor were further performed to confirm the biological function of miR-93 in glioma progression. As shown in Figure 3A, suppression of miR-93 by miR-93 inhibitor significantly decreased the growth rate of LN18 and Hs683 cells compared with that of NC transfected cells. The anchorage-independent growth assay revealed that miR-93-silenced cells produced fewer and smaller colonies than the negative control cells (Figure 3B). Furthermore, the level of DNA synthesis was significantly suppressed in miR-93-inhibitor transfected LN18 and Hs683 cells, whereas the control cells displayed relatively higher BrdUrd incorporation rates (Figure 3C). In addition, flow cytometry showed a significant increase in the percentage of cells in G1/G0 phase and a decrease in the percentage of cells in S phase in cells transfected with the miR-93 inhibitor compared with NC transfected cells (Figure 3D). These results suggest that downregulation of miR-93 could reduce the proliferation, tumorigenicity and cell cycle progression of glioma cells.

### miR-93 directly suppresses PTEN, PHLPP2 and FOXO3 in glioma cells

**Figure 4:**
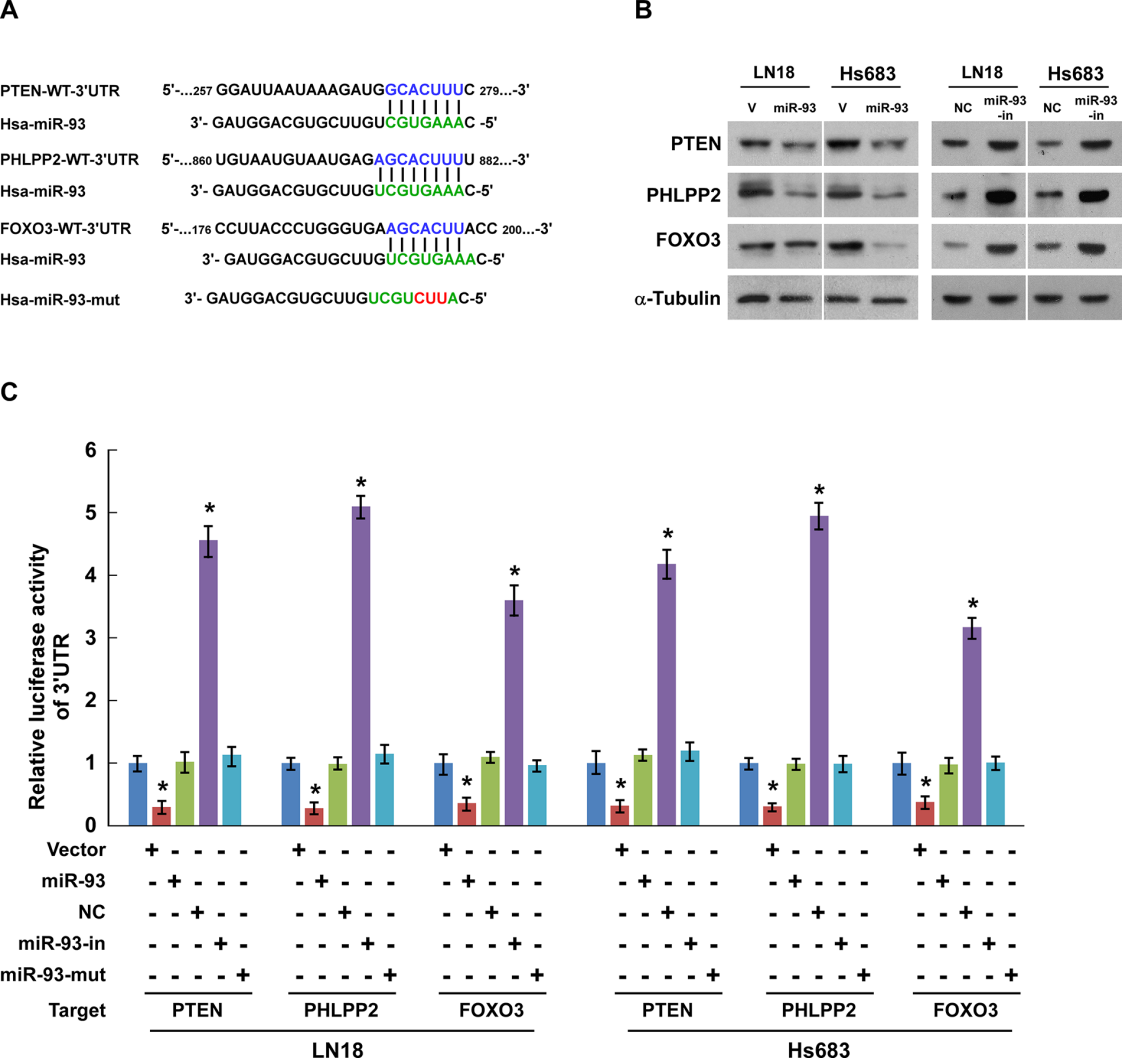
PTEN, PHLPP2, and FOXO3 are direct targets of miR-93 in glioma cells **(A)** Schematic putative target sites of miR-93 in 3′UTRs of PTEN, PHLPP2 and FOXO3, and the sequence of miR-93 mutant (performed as miR-93-mut). **(B)** Western blotting analysis of the protein levels of PTEN, PHLPP2 and FOXO3 in the indicated cells. **(C)** Luciferase assay of pGL3-PTEN, PHLPP2 or FOXO3–3′UTR reporter co-transfected with miR-93, miR-93 inhibitor or the control in the indicated cells. Experiments were repeated at least 3 times with similar results, and error bars represent ± SD, *P < 0.05.

In an attempt to identify the mRNA targets of miR-93, we performed a bioinformatics analysis using the publicly available algorithm (TargetScan 6.2). As shown in Figure 4A, PTEN and PHLPP2, which are the inhibitors of PI3K/Akt signaling pathway, and FOXO3, which critical regulator of cell-cycle, were found to be potential targets of miR-93. Western blotting analysis showed that ectopic expression of miR-93 dramatically decreased, whereas inhibition of miR-93 increased, the protein expression of PTEN, PHLPP2 and FOXO3 in both LN18 and Hs683 glioma cells (Figure 4B). Furthermore, when co-transfected with *PTEN*-, *PHLPP2*-, or *FOXO3*-3′UTR dual luciferase reporter plasmid, together with miR-93, miR-93 inhibitor or negative control, into the glioma cells, as shown in Figure 3C, miR-93 led to a consistent reduction of luciferase activity of *PTEN*, *PHLPP2*, *FOXO3* reporters. Whereas transfection with miR-93 inhibitor upregulated the luciferase activity, respectively (Figure 4C). Of note, the luciferase activity was insusceptible by miR-93-mut (miR-93 mutant) transfection instead of miR-93 (Figure 4C). Besides, the *PTEN*, *PHLPP2* or *FOXO3* 3′-UTR-luciferase reporter with a mutant miR-93 binding site seed sequence was not inhibited by ectopic expression of miR-93 (Supplementary Figure 2A and 2B). Taken together, the results confirm that *PTEN*, *PHLPP2* and *FOXO3* are direct targets of miR-93.

### PTEN, PHLPP2 or FOXO3 suppression is critical for miR-93-induced cell proliferation and tumorigenesis in glioma

**Figure 5:**
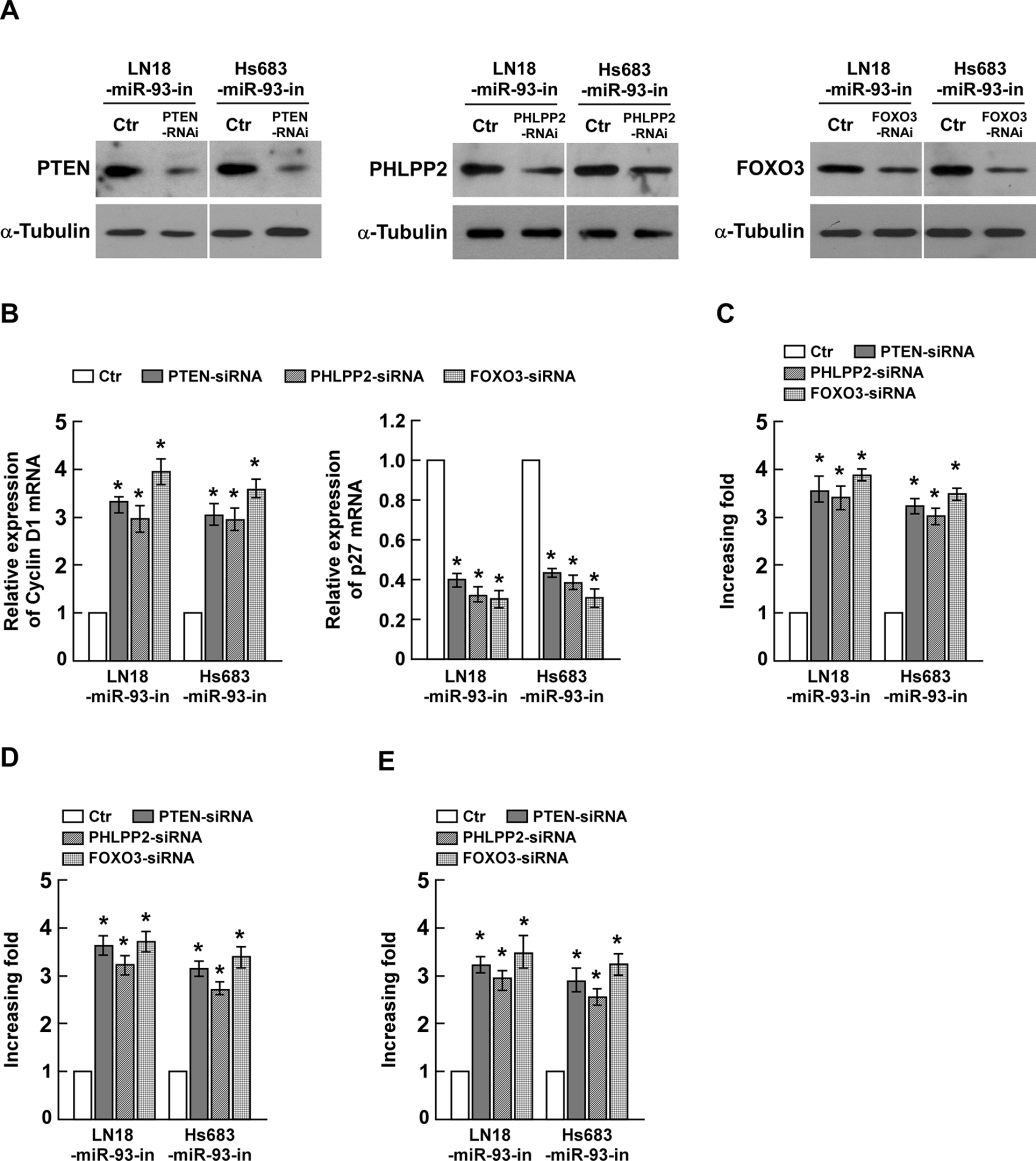
Suppression of PHLPP2, FOXO3, and PTEN by miR-93 is essential for glioma cell proliferation **(A)** Western blotting analysis of the protein levels of PTEN, PHLPP2 and FOXO3 in indicated cells transfected with specific siRNA, respectively. **(B)** The mRNA expression levels of Cyclin D1 and p27^Kip1^, determined by real-time PCR analysis. **(C)** Quantification of colonies determined by colony formation assay in indicated glioma cell lines. **(D)** Quantification of colonies determined by anchorage-independent growth ability assay in indicated glioma cell lines. **(E)** Quantification of BrdUrd incorporating-cells in indicated glioma cell lines. Experiments were repeated at least 3 times with similar results, and error bars represent ± SD. *P < 0.05.

To evaluate the effects of PTEN, PHLPP2 and FOXO3 on miR-93-induced glioma progression, we suppressed endogenous PTEN, PHLPP2 or FOXO3 expression with specific siRNAs (Figure 5A). As shown Figure 5B, silencing PTEN, PHLPP2 or FOXO3 in miR-93 inhibitor transfected cells increased the expression level of Cyclin D1 and decreased the expression of p27^Kip^. The colony formation assay indicated that silencing PTEN, PHLPP2 or FOXO3 in miR-93 inhibitor transfected cells increased proliferation of glioma cells (Figure 5C). The anchorage-independent growth assay and BrdUrd incorporation assay both showed the similar results (Figure 5D and 5E). These results suggested that silencing PTEN, PHLPP2 or FOXO3 expression in miR-93-repressed cells could reverse the inhibitory effect of the miR-93 inhibitor on glioma cell proliferation and tumorigenesis.

### Activated PI3K/Akt signaling is essential for miR-93-promoted glioma proliferation

**Figure 6:**
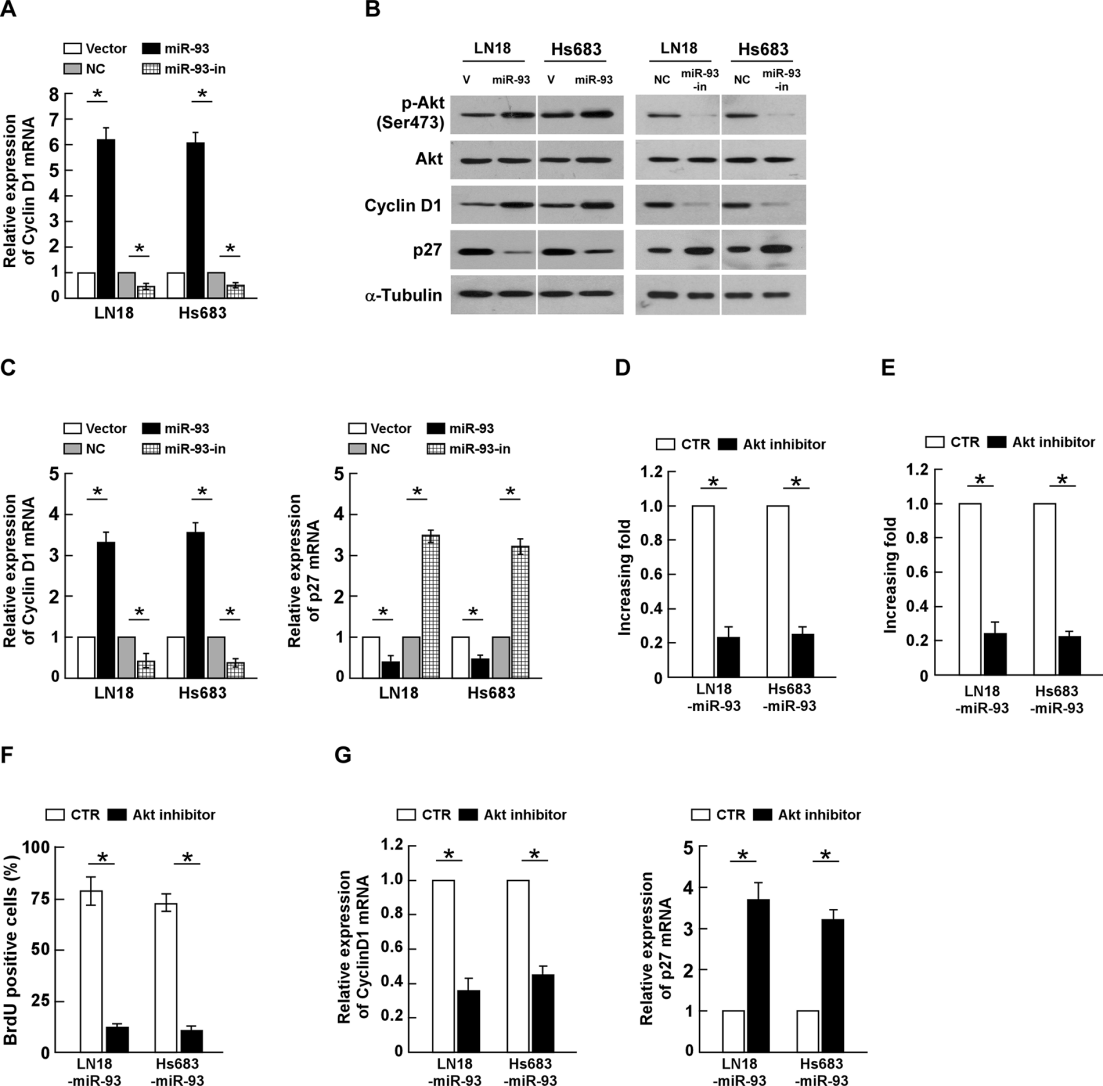
miR-93 activates PI3K/Akt signaling **(A)** Relative Akt activity in the indicated cells, determined by K-LISA Akt Activity assay. **(B)** Western blotting analysis of phosphorylated Akt (p-Akt) (Ser 473), total Akt, Cyclin D1 and p27^Kip1^ protein levels in indicated cells. α-Tubulin was used as a loading control. **(C)** The mRNA expression level of Cyclin D1 and p27^Kip1^, determined by real-time PCR analysis. **(D)** Quantification of colonies formed determined by colony formation assay in indicated glioma cell lines, treated with Akt inhibitor (0.5 μM). **(E)** Quantification of colonies determined by anchorage-independent growth ability assay in indicated glioma cell lines, treated with Akt inhibitor (0.5 μM). **(F)** Quantification of BrdUrd incorporating-cells in indicated glioma cell lines, treated with Akt inhibitor (0.5 μM). **(G)** The mRNA expression level of Cyclin D1 and p27^Kip1^, determined by real-time PCR analysis, in indicated glioma cell lines, treated with Akt inhibitor (0.5 μM). Experiments were repeated at least 3 times with similar results, and error bars represent ± SD. *P < 0.05.

It has been demonstrated that PTEN and PHLPP2 are critical negative regulators of PI3K/Akt signaling, which prompted us to further examine whether dysregulation miR-93 alters the activity of PI3K/Akt signaling in glioma cells. As shown in Figure 6A and 6B, overexpressing miR-93 significantly increased, but silencing miR-93 decreased, the Akt activity and the expression of phosphorylated Akt (Ser 473) in glioma cells. Consistently, the transcriptional and translational levels of Cyclin D1 and p27^kip1^, two downstream effectors of PI3K/Akt signaling, were also significantly alliterated in the miR-93-deregulated glioma cells (Figure 6B and 6C). These results indicate that miR-93 activates PI3K/Akt signaling. Furthermore, we examined whether activation of PI3K/Akt signaling contributed to miR-93-mediated gliomas cell proliferation. As shown in Figure 6D and 6E, inactivation of PI3K/Akt signaling using Akt inhibitor significantly decreased the growth rates of miR-93-transduced glioma cells, analyzed by colony formation and anchorage-independent growth assays. Moreover, the percentage of BrdUrd incorporating-cells and Cyclin D1 expression significantly decreased, but the expression of p27^kip1^ increased, in the miR-93-transduced cells treated with Akt inhibitor (Figure 6F and 6G). Taken together, our results demonstrate that PI3K/Akt signaling plays essential function during miR-93-induced glioma cells proliferation.

## DISCUSSION

**Figure 7:**
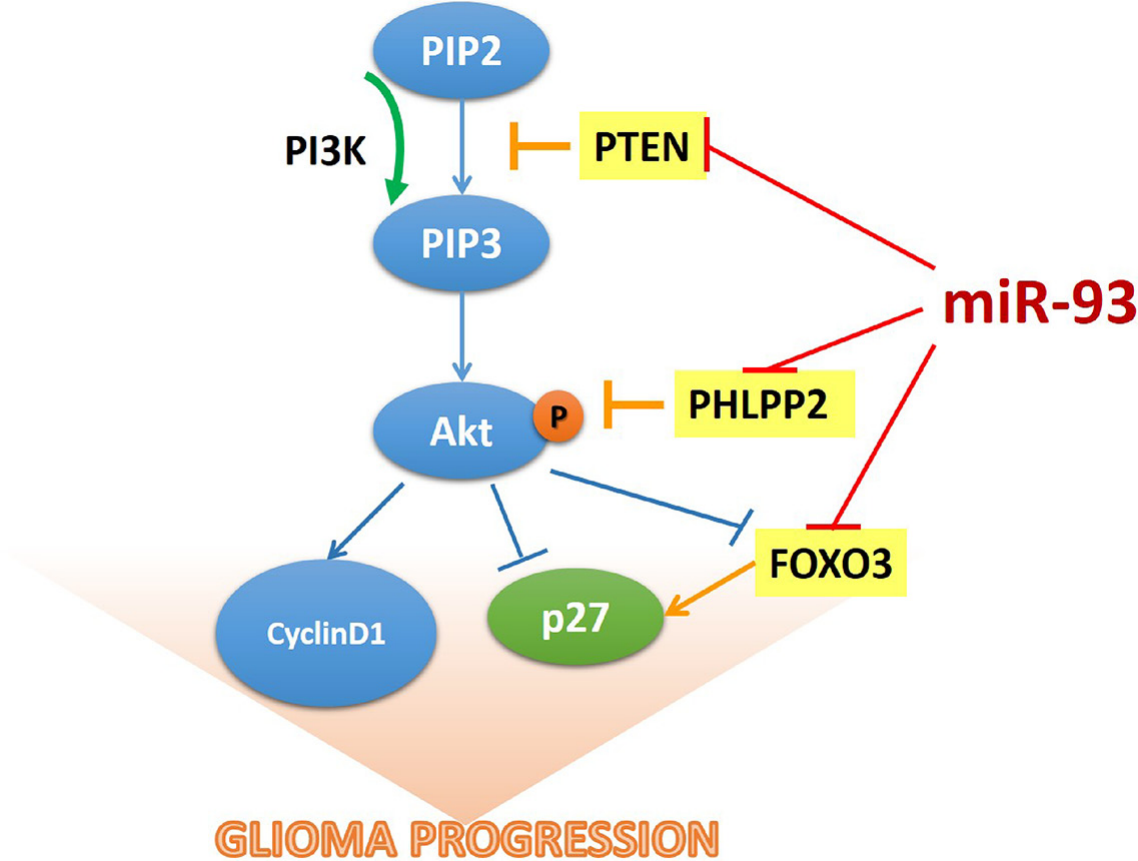
The model of miR-93-mediated PI3K/Akt signaling activation via down-regulation of PTEN, PHLPP2 and FOXO3 that results in the promotion of cell proliferation in gliomas

Numerous studies have demonstrated that activation of the PI3K/Akt signaling pathway is essential to the development and/or progression of most cancer types and associated with nearly all aspects of the malignant phenotype of cancer, such as uncontrolled proliferation, resistance to cell death, invasiveness, angiogenesis and metastasis [1, 6]. Inhibition of PI3K/Akt signaling, therefore, may represent a promising anti-cancer strategy [6]. Thus, a better understanding of molecular mechanism in which the PI3K/Akt signaling pathway is dysregulated in various cancers might facilitate development of specific targeting therapies. Herein, we found that miR-93 was significantly upregulated in gliomas, and overexpressing miR-93 activated PI3K/Akt signaling through downregulating PTEN, PHLPP2 and FOXO3 expression via targeting their 3′UTRs, subsequently resulting in glioma cell proliferation and progression (Figure 7). Therefore, our results suggested that miR-93 contributes to progression of glioma and might represent as a potential therapeutic target for glioma therapy.

Function as key PI3K/Akt inhibitor, PTEN has been found lost in multiple cancer types through gene deletion, mutation or epigenetic silencing during oncogenesis [36]. Genetic inactivation of PTEN, including mutation and deletion, occurs quite frequently in more than 50% of glioblastoma cases [19]. However, there is still a proportion of glioma cases that do not carry PTEN mutation and deletion, and the mechanism responsible for the abnormal PTEN expression in these patients remains largely unknown [37]. Meanwhile, the PHLPP2 gene resides at the chromosomal location 16q22.3, which is frequently lost in multiple cancer types. Loss of heterozygosity (LOH) has been observed at the PHLPP2 locus in breast cancer, ovarian cancers, prostate cancer, Wilms tumors and hepatocellular carcinomas [38]. Somatic mutation of PHLPP2, however, occurs quite rarely in human cancers. FOXO3, one member of subfamily of the forkhead transcription factors [39], is a potent transcriptional activator that triggers the expression of a program of genes involved in cell cycle arrest, apoptosis, and DNA repair and hypoxia response [39]. FOXO3 is known as a tumor suppressor, and deregulation of FOXO3 is involved in various tumor types [40–45]. It has been reported that FOXO3 plays a critical role in suppressing tumor growth by increasing cell cycle inhibitor p27^kip1^ [46–48]. Activated Akt leads to FOXO3 phosphorylation, and results in FOXO3 translocating from the nucleus to cytoplasm by binding with 14-3-3 chaperone protein, followed by FOXO3 degradation [49–52]. Hence, the activity of FOXO3 is closely related to the modulation of PI3K/Akt signaling. The inhibition of FOXO3 is usually due to high-level activation of multiple kinases in cancer, such as Akt and IκB kinase [51, 53]. In addition, the protein degradation mechanism, such as Ub-proteasome pathway, plays an essential role in regulating FOXO3 tumor suppressor function [45, 54]. Taken together, it appears that multiple molecular mechanisms are served to negatively regulate PI3K/Akt activity and maintain it at optimal levels, ensuring physiological cell survival but without over-proliferation or uncontrolled cell survival. In the scenario of cancer, therefore, for an over-activation of PI3K/Akt to occur, multiple inhibitors of the pathway in one cell need to be repressed in order the cell to overcome the redundant inhibition of PI3K/Akt signaling. In this context, our finding that a simultaneous suppression of three potent inhibitors of the PI3K/Akt pathway by miR-93 strongly demonstrates a unique significance of miRNA in modulating essential signaling pathways as it targets multiple genes. In the same context, such a multi-target effect of miR-93 on PI3K/Akt signaling also makes the miRNA molecule a potential target for delivery of effective and efficient inhibitory approaches against the pathway. Besides, PI3K/Akt signaling pathway is a multi-function pathway during tumor progression. The activation of PI3K-Akt pathway induces series of genes expression involved in tumor cell survival, proliferation, cell-cycle progression, migration, as well as angiogenesis [1–7]. Therefore, it would be interesting to further examine the potential functions of miR-93 on other aspects of glioma progression.

As an alternative mechanism to genetic alteration, overexpression of miR-93 might explain the observed over-activation of PI3K/Akt in glioma cells lack of PI3K amplification and genetic losses of PTEN, PHLPP2 and FOXO3. Activation of PI3K/Akt leads to upregulation of cell-cycle regulator Cyclin D1 and downregulation of p27^Kip1^, resulting in the promotion of glioma proliferation, cell cycle progression and a significant increase in the percentage of S-phase cell. These findings suggest that miR-93 functions as an onco-miR in glioma progression at the level of enhancing cell proliferation. Nevertheless, whether and how this miRNA molecule affects other aspects of the malignant phenotype of human glioma at different disease stages are needed to further investigated.

Through analyzed by TCGA database, we found that the genomic copy number of miR-93 was substantially upregulated in 82.19% of GBM patients (*n* = 529), suggesting that the upregulated miR-93 in gliomas might be caused by genomic amplification. Meanwhile, the miR-93 sequence is located in the intron of the *MCM7* (minichromosome maintenance 7) gene at chromosome 7q22.1. MCMs expression was significantly up-regulated in variety of tumors and the expression of MCM7 has been reported as an independent risk factor for relapse-free survival and correlated with poor outcome in patients with colorectal cancer, oesophageal squamous cell carcinoma and glioma [55–57]. Interestingly, we found that there are several putative binding sites of NF-κB p50 and p65 on the promoter of *MCM7*, which suggesting that NF-κB might be involved in the upregulation of miR-93 in glioma. Currently, the molecular mechanisms in which miR-93 is overexpressed in gliomas are under investigation in our laboratory.

## MATERIALS AND METHODS

### Cell culture and treatments

Normal human astrocytes (NHA) were purchased from the Sciencell Research Laboratories (Sciencell, Carlsbad, CA, USA) and cultured under the condition as the manufacturer instructed. Glioma cell lines U251MG, A172, LN229, SF767, U118MG, U87MG, Hs683, LN18 and SHG44 were grown in Dulbecco's modified Eagle's medium (DMEM, Invitrogen, Carlsbad, CA, USA) supplemented with 10% fetal bovine serum (FBS, Invitrogen), at 37°C in a 5% CO_2_ atmosphere in a humidified incubator. Akt inhibitor MK-2206 (1 μM, Selleck Chemicals, Houston, TX, USA) was used to treat cells according to the manufacturer's instructions.

### Tissue specimens and patient information

A total of 110 paraffin-embedded, archived glioma specimens and 10 freshly collected paired glioma tissues were histopathologically diagnosed at the First Affiliated Hospital of Sun Yat-sen University from 2000 to 2010. The clinicopathological characteristics of the samples are summarized in Supplementary Table 1. All samples were collected and analyzed with prior written informed consent from the patients. Normal brain tissues were obtained from individuals who died in traffic accidents and confirmed to be free of any pre-existing pathologically detectable conditions. Prior donors' consents and approvals from the Institutional Research Ethics Committee were obtained.

### Generation of stably engineered cell lines

pMSCV-miR-93 was generated by cloning the genomic pre-miR-93 gene with about 200-bp on each flanking side (primers used: forward, 5′-GCCAGATCTGCACTGTGGGTACTTGCTGC-3′; reverse, 5′-GCCGAATTCGC ACTGTGGGTACTTGCT GC-3′) into the retroviral transfer plasmid pMSCV-puro (Clontech Laboratories Inc., Mountain View, CA, USA). pMSCV-miR-93 was then cotransfected with the pIK packaging plasmid into 293FT cells, using the standard calcium phosphate transfection method [35]. Thirty-six hours after transfection, the supernatants were collected and then incubated with glioma cells to be infected for 24 hours in the presence of polybrene (2.5 μg/ml, Sigma). Puromycin (1.5 μg/ml, Sigma) was used to select stably transduced cells over 10 days after infection.

### RNA extraction and real-time quantitative PCR

Total cellular RNA was extracted using Trizol reagent (Invitrogen), according to the manufacturer's instruction. cDNAs were synthesized and real-time PCR was performed using the GoTaq^®^ 2-Step RT-qPCR System (Promega, Madison, WI, USA). SYBR Green I (Invitrogen) was used to quantify PCR amplification and the qRT-PCR was performed and analyzed using a 7500 Fast Real-Time Sequence detection system (Applied Biosystems, Foster City, CA, USA). miRNA quantification was determined by using Bulge-loop™ miRNA qRT-PCR Primer Set (one RT primer and a pair of qPCR primers for each set) specific for miR-93, designed by RiboBio (RiboBio Co. Ltd, Guangzhou, Guangdong, China). The expression of the miRNA was defined based on Ct, and relative expression levels were calculated as 2^−[(Ct of *miR-93*) – (Ct of *U6*)]^ after normalization with reference to the expression of small nuclear RNA U6. The extracted RNA was pretreated with RNase-free DNase, and 500 ng of RNA from each sample was used for cDNA synthesis primed with the specific microRNA RT-primer. For PCR amplification of cDNA, an initial amplification using primers was done with a denaturation step at 95°C for 20 seconds, followed by 40 cycles of denaturation at 95°C for 10 seconds, primer annealing at 60°C for 20 seconds, and primer extension at 70°C for 5 seconds. Expression levels of genes were normalized to that of the housekeeping gene GAPDH as the control and calculated as 2^−[(Ct of GENES) – (Ct of GAPDH)]^, where Ct represents the threshold cycle for each transcript. The following primers were used: *p27* forward, 5′-CATTCCATGAAGTCAGC GAT-3′, reverse, 5′-CGTCAA ACGTAAACAGCTCG-3′. *Cyclin D1* forward, 5′-AACTACC TGGACCGCTTCCT-3′, and reverse, 5′-CCACTTGAGCTTGTTCACCA-3′. *GAPDH* forward: 5′-GACTCATGACCACAGTCCATGC-3′, reverse: 3′-AGAGGCAGGGAT GATGTTCTG-5′.

### Plasmids, oligonucleotides, siRNA and transfection

The region of the human PTEN, PHLPP2, and FOXO3 3′-UTR, generated by PCR amplification from DNA of LN18 cells, was cloned into vector pGL3 (Promega). miR-93 inhibitor (miR-93 inhibitor is a LNA/OMe modified antisense oligonucleotide designed specifically to bind to and inhibit endogenous miR-93 molecule) and negative control were purchased from RiboBio. For depletion of PTEN, PHLPP2 and FOXO3, siRNAs were synthesized and purified by RiboBio. Transfection of oligonucleotides and siRNAs were performed using the Lipofectamine 2000 reagent (Invitrogen), according to the manufacturer's instruction.

### Western blotting analysis

Total protein was extracted from whole cells and 20 μg of isolated protein was separated by SDS-PAGE and electroblotted onto a PVDF membrane (Bio-Rad Laboratories, Hercules, CA, USA). The membranes were then probed with antibodies: anti-PTEN, anti-PHLPP2, anti-FOXO3, anti-Akt, anti-phospho-Akt, anti-Cyclin D1, or anti-p27 primary antibodies (Cell Signaling, Danvers, MA, USA). The membranes were stripped and reblotted with an anti-α-tubulin monoclonal antibody (Sigma) as a loading control.

### Colony formation assay

Cells were plated on 60mm-dish (1000 cells per dish) and cultured for 10 days. The colonies were stained with 0.1% crystal violet for 5 min after fixation with 10% formaldehyde for 15 min. Viable colonies that contained more than 50 cells were counted. The experiment was performed for three independently times for each cell line.

### Anchorage-independent growth ability assay

Cells (1 × 10^3^) were trypsinized and suspended in 2 ml complete medium plus 0.33% agar (Invitrogen) and plated in 6-well plate on top of a bottom agar layer (0.66% complete medium agar). After two-week days incubation, colony sizes were measured with an ocular micrometer and colonies greater than 0.1 mm in diameter were counted. All experiments were performed in triplicate.

### Bromodeoxyuridine labeling and immunofluorescence

Cells grown on coverslips (Fisher, Pittsburgh, PA, USA) were incubated with bromodeoxyuridine (BrdUrd) for 1 h and stained with anti-BrdUrd antibody (Sigma) according to the manufacturer's instruction. Gray level images were acquired under a laser scanning microscope (Axioskop 2 plus, Carl Zeiss Co. Ltd., Jena, Germany).

### Flow cytometry analysis

All cells in a culture dish were harvested by trypsinization, washed in ice-cold PBS, and fixed in 80% ice-cold ethanol in PBS. Before staining, the cells were spun down in a cooled centrifuge and resuspended in the cold PBS. Bovine pancreatic RNAase (Sigma) was added at a final concentration of 2 μg/ml, and cells were incubated at 37°C for 30 min, followed by incubation in 20 μg/ml of propidium iodide (Sigma) for 20 min at room temperature. Fifty thousand cells were analyzed flow cytometrically.

### Luciferase assay

Cells were seeded in triplicate in 24-well plate and allowed to settle for about 12 h. One hundred nanograms of pGL3-*PTEN*, -*PHLPP2* or -*FOXO3*-luciferase plasmid was co-transfected into glioma cells with TK-Renilla plasmid as control signals using the Lipofectamine 2000 reagent, according to the manufacturer's instruction. Luciferase and control signals were measured at 48 h after transfection using the Dual Luciferase Reporter Assay Kit (Promega, Madison, WI, USA), according to a protocol provided by the manufacturer. Three independent experiments were performed and the data were presented as the mean ± SD.

### Akt activity assay

Cells were serum-starved and treated with EGF (10 ng/ml) or insulin (100 nM) for 5 min. To measure kinase activities of in cells or tumor tissues, Akt was precipitated by a specific anti-Akt antibody. The immune-complexes were then incubated with a biotinylated peptide substrate, which became phosphorylated in the presence of activated Akt. The phosphorylated substrates, which reflected the activity of Akt kinase in the extract, was then quantified with the K-LISA Akt Activity Kit (Calbiochem, Darmstadt, Germany) that comprises a primary antibody recognizing the phosphorylated substrate peptides.

### Statistical analysis

Statistical tests for data analysis included Fisher's exact test, log-rank test, Chi-square test, and Student's 2-tailed *t* test. Bivariate correlations between study variables were calculated by Spearman's rank correlation coefficients. Survival curves were plotted by the Kaplan-Meier method and compared by the log-rank test. The significance of various variables for survival was analyzed by univariate and multivariate Cox regression analyses. Statistical analyses were performed using the SPSS 11.0 statistical software package. Data represent mean ± SD. *P* values of 0.05 or less were considered statistically significant.

## SUPPLEMENTARY INFORMATION



## Abbreviations

miR: microRNA
PTEN: Phosphatase and tensin homolog
PI3K: phosphatidylinositol-3-OH kinase
PIP3: Phosphatidylinositol 3, 4, 5-trisphosphate
PH: pleckstrin-homology
PHLPP: PH domain leucine rich repeat protein phosphatases
PDZ: PSD-95/Disc-large/ZO-1
IκB kinase: IKK; 3′UTR, 3′ untranslated region
NHA: normal human astrocytes
Mut: mutation

## References

[1] Fresno Vara JA, Casado E, de Castro J, Cejas P, Belda-Iniesta C, Gonzalez-Baron M (2004). PI3K/Akt signalling pathway and cancer. Cancer treatment reviews.

[2] Fruman DA, Meyers RE, Cantley LC (1998). Phosphoinositide kinases. Annual review of biochemistry.

[3] Alessi DR, Andjelkovic M, Caudwell B, Cron P, Morrice N, Cohen P, Hemmings BA (1996). Mechanism of activation of protein kinase B by insulin and IGF-1. The EMBO journal.

[4] Testa JR, Bellacosa A (2001). AKT plays a central role in tumorigenesis. Proc Natl Acad Sci U S A.

[5] Cheung M, Testa JR (2013). Diverse mechanisms of AKT pathway activation in human malignancy. Current cancer drug targets.

[6] Courtney KD, Corcoran RB, Engelman JA (2010). The PI3K pathway as drug target in human cancer. Journal of clinical oncology: official journal of the American Society of Clinical Oncology.

[7] Cheng GZ, Park S, Shu S, He L, Kong W, Zhang W, Yuan Z, Wang LH, Cheng JQ (2008). Advances of AKT pathway in human oncogenesis and as a target for anti-cancer drug discovery. Current cancer drug targets.

[8] Cappellini A, Tabellini G, Zweyer M, Bortul R, Tazzari PL, Billi AM, Fala F, Cocco L, Martelli AM (2003). The phosphoinositide 3-kinase/Akt pathway regulates cell cycle progression of HL60 human leukemia cells through cytoplasmic relocalization of the cyclin-dependent kinase inhibitor p27(Kip1) and control of cyclin D1 expression. Leukemia.

[9] Chen XL, Ren KH, He HW, Shao RG (2008). Involvement of PI3K/AKT/GSK3beta pathway in tetrandrine-induced G1 arrest and apoptosis. Cancer biology & therapy.

[10] Tanwar PS, Kaneko-Tarui T, Lee HJ, Zhang L, Teixeira JM (2013). PTEN loss and HOXA10 expression are associated with ovarian endometrioid adenocarcinoma differentiation and progression. Carcinogenesis.

[11] Markman B, Tao JJ, Scaltriti M (2013). PI3K pathway inhibitors: better not left alone. Curr Pharm Des.

[12] Patel R, Gao M, Ahmad I, Fleming J, Singh LB, Rai TS, McKie AB, Seywright M, Barnetson RJ, Edwards J, Sansom OJ, Leung HY (2013). Sprouty2, PTEN, and PP2A interact to regulate prostate cancer progression. J Clin Invest.

[13] Lumb CN, Sansom MS (2013). Defining the membrane-associated state of the PTEN tumor suppressor protein. Biophys J.

[14] Wu H, Goel V, Haluska FG (2003). PTEN signaling pathways in melanoma. Oncogene.

[15] Bostrom J, Cobbers JM, Wolter M, Tabatabai G, Weber RG, Lichter P, Collins VP, Reifenberger G (1998). Mutation of the PTEN (MMAC1) tumor suppressor gene in a subset of glioblastomas but not in meningiomas with loss of chromosome arm 10q. Cancer Res.

[16] Chiariello E, Roz L, Albarosa R, Magnani I, Finocchiaro G (1998). PTEN/MMAC1 mutations in primary glioblastomas and short-term cultures of malignant gliomas. Oncogene.

[17] Duerr EM, Rollbrocker B, Hayashi Y, Peters N, Meyer-Puttlitz B, Louis DN, Schramm J, Wiestler OD, Parsons R, Eng C, von Deimling A (1998). PTEN mutations in gliomas and glioneuronal tumors. Oncogene.

[18] Figarella-Branger D, Colin C, Coulibaly B, Quilichini B, Maues De Paula A, Fernandez C, Bouvier C (2008). Histological and molecular classification of gliomas. Revue neurologique.

[19] Song MS, Carracedo A, Salmena L, Song SJ, Egia A, Malumbres M, Pandolfi PP (2011). Nuclear PTEN regulates the APC-CDH1 tumor-suppressive complex in a phosphatase-independent manner. Cell.

[20] Brognard J, Niederst M, Reyes G, Warfel N, Newton AC (2009). Common polymorphism in the phosphatase PHLPP2 results in reduced regulation of Akt and protein kinase C. J Biol Chem.

[21] Brognard J, Sierecki E, Gao T, Newton AC (2007). PHLPP and a second isoform, PHLPP2, differentially attenuate the amplitude of Akt signaling by regulating distinct Akt isoforms. Molecular cell.

[22] Ambros V (2004). The functions of animal microRNAs. Nature.

[23] Bartel DP (2004). MicroRNAs: genomics, biogenesis, mechanism, and function. Cell.

[24] Ruvkun G (2006). Clarifications on miRNA and cancer. Science.

[25] Calin GA, Croce CM (2006). MicroRNA signatures in human cancers. Nat Rev Cancer.

[26] Cimmino A, Calin GA, Fabbri M, Iorio MV, Ferracin M, Shimizu M, Wojcik SE, Aqeilan RI, Zupo S, Dono M, Rassenti L, Alder H, Volinia S, Liu CG, Kipps TJ, Negrini M, Croce CM (2005). miR-15 and miR-16 induce apoptosis by targeting BCL2. Proc Natl Acad Sci U S A.

[27] Godlewski J, Nowicki MO, Bronisz A, Williams S, Otsuki A, Nuovo G, Raychaudhury A, Newton HB, Chiocca EA, Lawler S (2008). Targeting of the Bmi-1 oncogene/stem cell renewal factor by microRNA-128 inhibits glioma proliferation and self-renewal. Cancer Res.

[28] Cho WC (2007). OncomiRs: the discovery and progress of microRNAs in cancers. Mol Cancer.

[29] Lyu X, Fang W, Cai L, Zheng H, Ye Y, Zhang L, Li J, Peng H, Cho WC, Wang E, Marincola FM, Yao K, Cai H, Li J, Li X (2014). TGFbetaR2 is a major target of miR-93 in nasopharyngeal carcinoma aggressiveness. Mol Cancer.

[30] Deng ZQ, Qian J, Liu FQ, Lin J, Shao R, Yin JY, Tang Q, Zhang M, He L (2014). Expression level of miR-93 in formalin-fixed paraffin-embedded tissues of breast cancer patients. Genetic testing and molecular biomarkers.

[31] Zhu W, He J, Chen D, Zhang B, Xu L, Ma H, Liu X, Zhang Y, Le H (2014). Expression of miR-2c, miR-3, and miR-42 as potential biomarkers for detection of early stage non-small lung cancer. PLoS One.

[32] Du L, Zhao Z, Ma X, Hsiao TH, Chen Y, Young E, Suraokar M, Wistuba I, Minna JD, Pertsemlidis A (2014). miR-93-directed downregulation of DAB2 defines a novel oncogenic pathway in lung cancer. Oncogene.

[33] Fang L, Du WW, Yang W, Rutnam ZJ, Peng C, Li H, O'Malley YQ, Askeland RW, Sugg S, Liu M, Mehta T, Deng Z, Yang BB (2012). MiR-93 enhances angiogenesis and metastasis by targeting LATS2. Cell cycle.

[34] Xu D, He XX, Chang Y, Sun SZ, Xu CR, Lin JS (2012). Downregulation of MiR-93 expression reduces cell proliferation and clonogenicity of HepG2 cells. Hepato-gastroenterology.

[35] Hahn WC, Dessain SK, Brooks MW, King JE, Elenbaas B, Sabatini DM, DeCaprio JA, Weinberg RA (2002). Enumeration of the simian virus 40 early region elements necessary for human cell transformation. Mol Cell Biol.

[36] Sarker D, Reid AH, Yap TA, de Bono JS (2009). Targeting the PI3K/AKT pathway for the treatment of prostate cancer. Clin Cancer Res.

[37] Cheng CK, Fan QW, Weiss WA (2009). PI3K signaling in glioma—animal models and therapeutic challenges. Brain Pathol.

[38] Brognard J, Newton AC (2008). PHLiPPing the switch on Akt and protein kinase C signaling. Trends Endocrinol Metab.

[39] Zhang Y, Gan B, Liu D, Paik JH (2011). FoxO family members in cancer. Cancer biology & therapy.

[40] Sobolesky PM, Halushka PV, Garrett-Mayer E, Smith MT, Moussa O (2014). Regulation of the tumor suppressor FOXO3 by the thromboxane-A2 receptors in urothelial cancer. PLoS One.

[41] Cho EC, Kuo ML, Liu X, Yang L, Hsieh YC, Wang J, Cheng Y, Yen Y (2014). Tumor suppressor FOXO3 regulates ribonucleotide reductase subunit RRM2B and impacts on survival of cancer patients. Oncotarget.

[42] Tikhanovich I, Kuravi S, Campbell RV, Kharbanda KK, Artigues A, Villar MT, Weinman SA (2014). Regulation of FOXO3 by phosphorylation and methylation in hepatitis C virus infection and alcohol exposure. Hepatology.

[43] Qi W, Fitchev PS, Cornwell ML, Greenberg J, Cabe M, Weber CR, Roy HK, Crawford SE, Savkovic SD (2013). FOXO3 growth inhibition of colonic cells is dependent on intraepithelial lipid droplet density. J Biol Chem.

[44] Chung YM, Park SH, Tsai WB, Wang SY, Ikeda MA, Berek JS, Chen DJ, Hu MC (2012). FOXO3 signalling links ATM to the p53 apoptotic pathway following DNA damage. Nature communications.

[45] Tsai WB, Chung YM, Zou Y, Park SH, Xu Z, Nakayama K, Lin SH, Hu MC (2010). Inhibition of FOXO3 tumor suppressor function by betaTrCP1 through ubiquitin-mediated degradation in a tumor mouse model. PLoS One.

[46] Stahl M, Dijkers PF, Kops GJ, Lens SM, Coffer PJ, Burgering BM, Medema RH (2002). The forkhead transcription factor FoxO regulates transcription of p27Kip1 and Bim in response to IL-2. Journal of immunology.

[47] Park KW, Kim DH, You HJ, Sir JJ, Jeon SI, Youn SW, Yang HM, Skurk C, Park YB, Walsh K, Kim HS (2005). Activated forkhead transcription factor inhibits neointimal hyperplasia after angioplasty through induction of p27. Arteriosclerosis, thrombosis, and vascular biology.

[48] Chandramohan V, Jeay S, Pianetti S, Sonenshein GE (2004). Reciprocal control of Forkhead box O 3a and c-Myc via the phosphatidylinositol 3-kinase pathway coordinately regulates p27Kip1 levels. Journal of immunology.

[49] Tran H, Brunet A, Griffith EC, Greenberg ME (2003). The many forks in FOXO's road. Sci STKE.

[50] Plas DR, Thompson CB (2003). Akt activation promotes degradation of tuberin and FOXO3a via the proteasome. J Biol Chem.

[51] Chapuis N, Park S, Leotoing L, Tamburini J, Verdier F, Bardet V, Green AS, Willems L, Agou F, Ifrah N, Dreyfus F, Bismuth G, Baud V, Lacombe C, Mayeux P, Bouscary D (2010). IkappaB kinase overcomes PI3K/Akt and ERK/MAPK to control FOXO3a activity in acute myeloid leukemia. Blood.

[52] Yamaguchi H, Hsu JL, Hung MC (2012). Regulation of ubiquitination-mediated protein degradation by survival kinases in cancer. Frontiers in oncology.

[53] Lv Y, Song S, Zhang K, Gao H, Ma R (2013). CHIP regulates AKT/FoxO/Bim signaling in MCF7 and MCF10A cells. PLoS One.

[54] Vogt PK, Jiang H, Aoki M (2005). Triple layer control: phosphorylation, acetylation and ubiquitination of FOXO proteins. Cell Cycle.

[55] Ishibashi Y, Kinugasa T, Akagi Y, Ohchi T, Gotanda Y, Tanaka N, Fujino S, Yuge K, Kibe S, Yoshida N, Mizobe T, Oka Y, Yoshida T, Shirouzu K (2014). Minichromosome maintenance protein 7 is a risk factor for recurrence in patients with Dukes C colorectal cancer. Anticancer research.

[56] Zhong X, Chen X, Guan X, Zhang H, Ma Y, Zhang S, Wang E, Zhang L, Han Y (2014). Overexpression of G9a and MCM7 in oesophageal squamous cell carcinoma is associated with poor prognosis. Histopathology.

[57] Hua C, Zhao G, Li Y, Bie L (2014). Minichromosome Maintenance (MCM) Family as potential diagnostic and prognostic tumor markers for human gliomas. BMC Cancer.

