# Supplementary material for: miR-93 Promotes Cell Proliferation in Gliomas through Activation of PI3K/Akt Signaling Pathway
Source: Oncotarget. 2015 Mar 13;6(10):8286–99. doi: 10.18632/oncotarget.3221 (PMC4480752; doi:10.18632/oncotarget.3221)
Supplement: Supplementary file 1 [file oncotarget-06-8286-s001.pdf]

SUPPLEMENTARY INFORMATION

The primers used for plasmid construction were

PTEN-3′UTR-wt forward: 5′-GCCCCGCG GAAGAGGGATAAAACACCATG-3′; PTEN-3′UTR-wt reverse: 3′-GCCCTGCAGAGGGGTAGGATGT GAACCAG-5′. PTEN-3′UTR-mut forward: 5′-CTCCA CTTTTTATAAAACTGGAATAAAACGGGTTTGTGCC ATCTTTA TTAATCCTAATTGAATTTTA-3′; PTEN -3′UTR-mut reverse: 5′-TAAAATTCAATTAGGA TTAAT AAAGATGGCACAAACCCGTTTTATTCCAGTTTTA TAAAAAGTGGAG-3′. PHLPP2-3′UTR-wt forward: 5′-GCCCCGCGGTGGCTCTGGATAAACTGGTA-3′; PHLPP2-3′UTR-wt reverse: 3′-GCCCTGCAGGCTG CCTCTAAGAAGATTGG-5′. PHLPP2–3′UTR-mut forward: 5′-CAGATT CAAAAATAAAAAGTTCAC AGTCA ATGATTTGTGCTCTCATTACATTACATAC ATAAATTATCT-3′; PHLPP2-3′UTR-mut reverse: 5′-AGATAATTTATGTATGTAATGTAATGAGAGCACAA ATCATTGAC TGTGAACTTTTTATTTTTGAATCTG -3′. FOXO3-3′UTR-wt forward: 5′-GCCCCGCGGAGCA AGTGGACAGTGATACC-3′; FOXO3-3′UTR-wt reverse: 3′-GCCCTGCAGAACTCAGAAAGTCAAAGGAA-5′. FOXO3-3′UTR-mut forward: 5′-TGTTCCAAGGGTTTT TGCTTCACCCAGGGTAAGGGGCC-3′; FOXO3 -3′UTR-mut reverse: 5′-GGCCCCTTACCCTGGGTGA AGCAAAAACCCTTGGAACA-3′.

SUPPLEMENTARY FIGURES AND TABLES

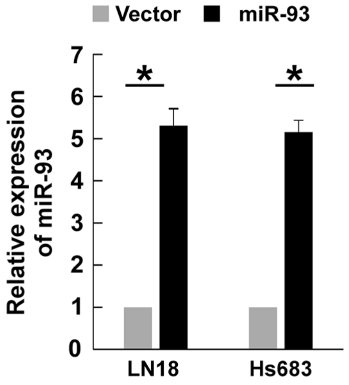

Supplementary Figure 1: Real-time PCR analysis of miR-93 expression in glioma cells stably expressing miR-93 or vector control. Transcript levels were normalized by *U6* expression. Experiments were repeated at least 3 times with similar results, and error bars represent ± SD. **P* < 0.05.

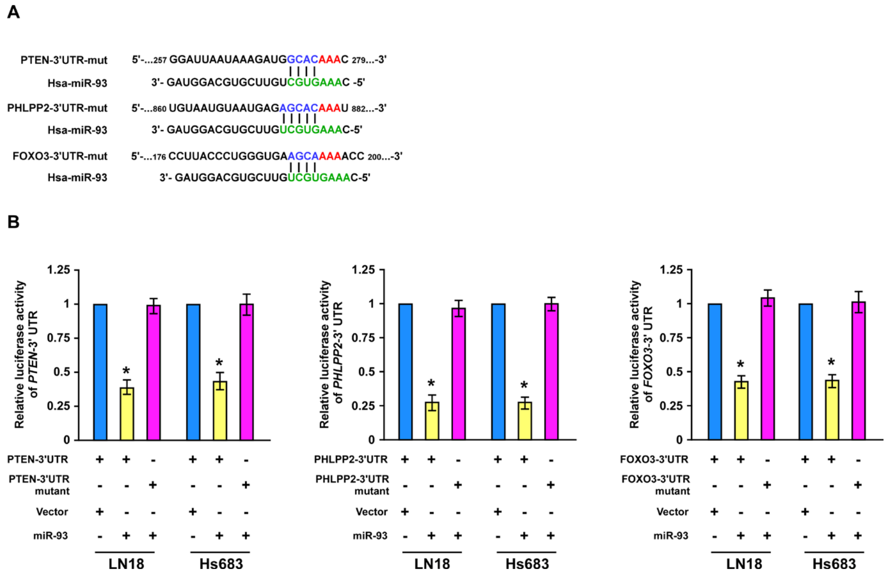

Supplementary Figure 2: PTEN, PHLPP2, and FOXO3 are direct targets of miR-93 in glioma cells. (A) Schematic putative target sites of miR-93 in 3′UTRs of PTEN, PHLPP2 and FOXO3, and the sequence of miR-93 mutant (performed as miR-93-mut). **(B)** Luciferase assay of pGL3- PTEN, PHLPP2 or FOXO3–3′UTR reporter with a mutant miR-93 binding site seed sequence co-transfected with miR-93 or the control in the indicated cells. Experiments were repeated at least 3 times with similar results, and error bars represent ± SD, **P* < 0.05.

Supplementary Table 1: Clinicopathological characteristics of studied patients and expression of miR-93 in glioma

Factor	No.	(%)
**Age (years)**		
≤ 45	70	63.6
> 45	40	36.4
**Gender**		
Male	79	71.8
Female	31	28.2
**Clinical stage**		
I	11	10
II	39	35.5
III	41	37.3
IV	19	17.3
**Vital status**		
Alive	33	30
Dead	77	70
**Expression of miR-93**		
Low expression	55	50
High expression	55	50

Supplementary Table 2: Correlation between the clinicopathological features and expression of miR-93 in glioma

Patient characteristics		miR-93 expression		*P*-value
		Low	High	
**Gender**	Male	44	35	0.056
	Female	11	20	
**Age (years)**	≤ 45	33	37	0.428
	> 45	22	18	
**Clinical stage**	I	8	3	< 0.001
	II	32	7	
	III	12	29	
	IV	3	16	
**Survival time (Median = 28.5 month)**	≤ 28.5	12	43	< 0.001
	> 28.5	43	12	
**Vital status**	Alive	24	9	0.002
	Dead	31	46	

Supplementary Table 3: Univariate and multivariate analysis of different prognostic parameters in patients with glioma by Cox-regression analysis

	No.	Univariate analysis		Multivariate analysis		
		P-Value	Regression coefficient (SE)	*P*-Value	Relative risk	95% confidence interval
**Age**						
≤ 45	70	< 0.001	1.033	0.007	1.011	0.996–1.026
> 45	40					
**Glioma histology (WHO grade)**						
I	11	< 0.001	4.020	< 0.001	3.283	2.222–4.852
II	39					
III	41					
IV	19					
**Expression of miR-93**						
Low	55	< 0.001	3.799	0.001	2.358	1.445–3.847
High	55					
